# Comparative Transcriptome Analysis Reveals Candidate Genes and Pathways for Potential Branch Growth in Elm (*Ulmus pumila*) Cultivars

**DOI:** 10.3390/biology11050711

**Published:** 2022-05-06

**Authors:** Luoyan Zhang, Shaoqiu Xie, Cheng Yang, Dongling Cao, Shoujin Fan, Xuejie Zhang

**Affiliations:** Key Lab of Plant Stress Research, College of Life Science, Shandong Normal University, Jinan 250014, China; zhangluoyan@sdnu.edu.cn (L.Z.); 2019020746@stu.sdnu.edu.cn (S.X.); 2020020799@stu.sdnu.edu.cn (C.Y.); 2019010082@stu.sdnu.edu.cn (D.C.)

**Keywords:** *Ulmus pumila*, branch thickening, comparative transcriptome, cultivars, xylan synthesis

## Abstract

**Simple Summary:**

Elm (*Ulmus pumila*) is a strong essential wood, and it is widely used in cabinets, sculptures, and ship making. In the present study, phenotypic and comparative transcriptomic analyses were performed in elm fast- (UGu17 and UZuantian) and slow-growing cultivars (U81-07 and U82-39). Phenotypic observation showed that the thickness of secondary xylem of 2-year-old fast-growing branches was greater compared with slow-growing cultivars. Comparative transcriptome analysis predicted that many pathways were involved in vascular development and transcriptional regulation in elm, such as “plant-type secondary cell wall biogenesis”, “cell wall thickening”, and “phenylpropanoid biosynthesis”. NAC domain transcriptional factors (TFs) and their master regulators, cellulose synthase catalytic subunits (CESAs), xylan synthesis, and secondary wall thickness were presumed to function in the thickening mechanism of elm branches. Our results indicated that the general phenylpropanoid pathway and lignin metabolism had vital functions in the growth of elm branches.

**Abstract:**

Wood plays a vital role in human life. It is important to study the thickening mechanism of tree branches and explore the mechanism of wood formation. Elm (*Ulmus pumila*) is a strong essential wood, and it is widely used in cabinets, sculptures, and ship making. In the present study, phenotypic and comparative transcriptomic analyses were performed in *U. pumila* fast- (UGu17 and UZuantian) and slow-growing cultivars (U81-07 and U82-39). Phenotypic observation showed that the thickness of secondary xylem of 2-year-old fast-growing branches was greater compared with slow-growing cultivars. A total of 9367 (up = 4363, down = 5004), 7159 (3413/3746), 7436 (3566/3870), and 5707 (2719/2988) differentially expressed genes (DEGs) were identified between fast- and slow-growing cultivars. Moreover, GO and KEGG enrichment analyses predicted that many pathways were involved in vascular development and transcriptional regulation in elm, such as “plant-type secondary cell wall biogenesis”, “cell wall thickening”, and “phenylpropanoid biosynthesis”. NAC domain transcriptional factors (TFs) and their master regulators (*VND1*/*MYB26*), cellulose synthase catalytic subunits (CESAs) (such as *IRX5*/*IRX3*/*IRX1*), xylan synthesis, and secondary wall thickness (such as *IRX9*/*IRX10*/*IRX8*) were supposed to function in the thickening mechanism of elm branches. Our results indicated that the general phenylpropanoid pathway (such as *PAL*/*C4H*/*4CL*) and lignin metabolism (such as *HCL*/*CSE*/*CCoAOMT*/*CCR*/*F5H*) had vital functions in the growth of elm branches. Our transcriptome data were consistent with molecular results for branch thickening in elm cultivars.

## 1. Introduction

Wood plays a vital role in human life. Humans have used wood for fuel, building materials, furniture, paper, tools and more. The thickness of branches is a key wood trait for the selection of trees with enhanced glucose yield and biomass production [1]. Previous studies have unraveled the links between wood quality and thickness of branches by studying populations of natural variants and genotypes [1,2]. Different cultivars or genotypes of the same tree have great differences in thickness due to environmental and genetic factors. In connection with the evaluation of wood quality, it is significantly necessary to study the thickening mechanism of tree branches and explore the formation mechanism of wood. The formation of vascular tissue is the most important process in the growth of tree branches. As physical support for upright growth, vascular tissues play a critical role in conveying water and nutrients throughout the plant [3,4,5].

The vascular cambium—a secondary lateral meristem—contains the stem cells and transient amplifying cells that produce the secondary phloem and secondary xylem (wood) [1]. Cambial activity is highly plastic throughout a plant’s life; cell division, expansion rates, cell-type specification, and differentiation in the cambium can all be varied in response to environmental and developmental factors [1]. As a complex process, vascular development of plants culminates in the generation of xylem and phloem, the transporting conduits of plants [6,7,8,9]. Xylem cells can develop into secondary cell walls (SCWs), which form the biggest section of plant lignocellulosic biomass serving as a renewable resource for biofuel production [6,10]. Biochemical, molecular, and genetic investigations have discovered that a large number of genes are involved in the biosynthesis of SCW components [11,12]. Cellulose synthase complexes synthesize cellulose in the plasma membrane [13]. Three Arabidopsis SCW CESA genes, *CESA4/IRX5*, *CESA7/IRX3*, and *CESA8/IRX1*, play significant roles in plant development, mutation of which leads to a seriously reduced cellulose amount and SCW thickness, resulting in decreased stem structural strength and an abnormal xylem phenotype [14,15,16,17]. Xylan, including its β-1,4-xylan backbone, glycosyl substituents, and acetyl groups, is produced in the Golgi apparatus, which are then released into the cell walls through vesicles. Genetic and biochemical studies have shown that the synthesis of the β-1,4-xylan backbone is regulated by a xylan synthase complex, and such a complex consists of a family GT47 proteins (IRX10/IRX10L) and two functionally nonredundant groups of family GT43 proteins (IRX9/IRX9L and IRX14/IRX14L) [18,19,20,21].

Plant hormonal response, transcriptional regulation, and peptide signaling control procambium/cambium proliferation, vascular patterning, and xylem differentiation [22,23]. Auxin is instrumental in regulating cambial stem cells’ activity and the differentiation of their derivatives. Studies of cryotome sections across wood forming tissues from poplar and spruce indicated that auxin concentrations are highest in the cambial zone [24,25] and decrease gradually upon moving away from the cambium toward the secondary phloem or the xylem. Independent proteomics and transcriptomics studies of *Pinus* sp. have tentatively identified ethylene as a potential regulator of the transition from juvenile to mature wood [26,27]. Both hierarchical and non-hierarchical regulatory networks are involved in the development of vascular tissues, which are regulated by many transcriptional factors (TFs) [6,28,29]. The biosynthetic genes of SCW are directly modulated by three layers of regulators, such as NAC domain master regulators (including NST1-3 and VND1-7) in tier three [30,31,32], two MYB domain regulators in tier two (as MYB46 and MYB83), and many other regulators in tier one [3,29]. Repression of these regulators reduces the thickness of the cell wall. By contrast, several genes responsible for the synthesis of cellulose, hemicellulose, and lignin, such as CesA8, IRX9, and 4CL, can be up-regulated once the TFs are over-expressed (like *MYB52* and *MYB54*) [33]. Besides, phenylpropanoid metabolism contributes to plant development, including vascular tissue thickening [34]. Lignin is synthesized through the phenylalanine/tyrosine metabolic pathway, which can increase cell wall rigidity and hydrophobic properties and enhance mineral delivery via the vascular bundles.

As a strong essential wood from the broadleaf family, Elm (*Ulmus pumila*) is a deciduous tree species belonging to the botanical classification of Ulmaceae, and is native to central Asia and distributed diffusely in Asia, America, and southern Europe [35,36,37]. The color of the wood is brownish–brown or reddish–yellow. A characteristic tone of the elm is that it darkens with the passage of time, giving it an added beauty. It is very hard, compact, rigid, and resistant and can be turned and carved. Currently, it is widely used in cabinet making, in the manufacture of sculptures, and even on ships [35,38]. In recent years, some transcriptome sequencing studies have been reported on the molecular mechanism of fruit development and salt tolerance of elm [36,39]. However, as an excellent wood, there is still no research on the mechanism of wood formation among different cultivars. Therefore, the aim of the present study was to reveal the mechanism of wood formation and its quality in elm cultivars on the basis of phenotypic and comparative transcriptomic analyses in fast- and slow-growing cultivars. To fully understand the branch growth in elm cultivars, it is necessary to discover candidate genes and potent pathways associated with vascular development and transcriptional regulation.

## 2. Materials and Methods

### 2.1. Plant Materials and Anatomical Observation

The material for the study was collected from Baiwa Forestry Centre, Shandong Province, China (35°2′22″ N, 116°8′22″ E), established in 1959, with a forest land area of 1.47 square kilometers. It is the only elm germplasm and gene bank in China where elm germplasm/cultivar resources have been collected and preserved from all over China.

In this study, 2-year-old seedlings of 50 *U. pumila* cultivars from the Baiwa Forestry Centre were selected for morphological analyses. The plant materials were cultured in natural conditions, and the branches were measured and collected in the vegetation season (21 June 2020). The measurement of branch diameters was performed by the method described by Wang [40]. At sampling time, for each cultivar, three trees in good condition were used for the measurements. Three branches were measured randomly from the selected tree (Figure S1b). Basal diameter (BD) of the internode was determined as the geometric mean of two measurements taken in perpendicular directions to 0.1 mm with digital calipers at the midpoint of the internode (Figure S1b). The growth rate of cultivars was estimated by relative growth rates (RGR): RGR = (lnN2 − lnN1)/Δt, where N1 is the BD at time-point 1 (16 March 2020) and N2 is BD at time-point 2 (21 June 2020), Δt is the time interval (months).

For anatomical observation, 10 cross-sections were excised from 10 branches collected from the fast- (UGu17 and UZuantian) and slow-growing cultivars (U81-07 and U82-39) and fixed in formalin-alcohol-acetic acid (FAA) for anatomical observation. The freehand slices of cross-sections were stained with phloroglucinol, and observed under a ZEISS Stemi 508 dissecting microscope (Germany) equipped with a computer-assisted digital camera.

### 2.2. RNA Extraction

UGu17 (Fast1, F1) and UZuantian (Fast2, F2) were selected as representative cultivars of the group with a fast branch growth rate, U81-07 (Slow1, S1) and U82-39 (Slow2, S2) were used as cultivars with a slow growth rate. At sampling time, for each cultivar, 9 small blocks of vascular tissue were excised from 9 branches collected from three different trees and fixed. A total of 3 replicates of blocks of vascular tissue were sampled for RNA extraction and transcriptome sequencing. For each replicate, a total of 1g plant materials were ground in liquid nitrogen and total RNAs were extracted using the TRIzol Reagent (Invitrogen, Carlsbad, CA, USA) following the manufacturer’s procedures. RNA quality was assessed using the RNA Nano 6000 Assay Kit of the Agilent Bioanalyzer 2100 system (Agilent Technologies, Santa Clara, CA, USA) and the NanoDrop 2000 spectrophotometer (Thermo Scientific, Wilmington, NC, USA).

### 2.3. Illumina Library Construction and Sequencing

Sequencing libraries were generated using the NEBNext^®^ UltraTM RNA Library Prep Kit for Illumina^®^ (NEB, San Diego, CA, USA) by following manufacturer’s procedures and index codes were added to attribute sequences to each sample. Briefly, mRNA was purified from total RNA using poly-T oligo-attached magnetic beads. After first and second strand cDNA synthesis, 150–200 bp cDNA fragments were purified with the AMPure XP system (Beckman Coulter, Indianapolis, IN, USA). The size-selected, adaptor-ligated fragments were purified and enriched by PCR amplification. The resulting products were used for sequencing analysis. High-throughput sequencing was conducted using an Illumina HiSeq X platform (Illumina, San Diego, CA, USA), according to the manufacturer’s procedures. All genetic data have been submitted to the NCBI Sequence Read Archive (SRA) database (https://www.ncbi.nlm.nih.gov/sra, accessed on 1 May 2022), SRA accession: PRJNA785411.

### 2.4. De Novo Assembly of Transcriptome

RNA sequencing and de novo transcriptome assembly were conducted to create reference sequence libraries for *U. pumila* branches. The RNA sample of every accession was sequenced separately. cDNA library construction and Illumina pair-end 150 bp sequencing (PE150) were performed according to instructions provided by Illumina Inc. Clean reads were obtained by removing reads containing adapters, reads containing ploy-N and low-quality row reads. The remaining high-quality reads were used for transcriptome assembly using the Trinity software pipeline with default parameters [41]. De novo assembled unigene sequences were used for BLAST searches and annotation against public databases (NR, NT, Swiss-Prot, Pfam, KOG/COG, Swiss-Prot, KEGG Ortholog database and Gene Ontology) with an E-value threshold of 1 × 10^−5^.

### 2.5. Calculation of Gene Expression Levels

Twelve independent transcripts libraries were generated for of *U. pumila* branches by a PE150 sequencing analysis, separately. Gene expression levels were estimated by RSEM [42] for all samples. The clean reads were aligned to the de novo assembled transcriptome. Gene expression levels of branch samples from *U. pumila* were calculated by the fragment per kilobase of exon model per million mapped reads (FPKM) method [43]. FPKM values were chosen to compare the expression levels between fast- and slow samples with a cutoff of adjusted *p*-value < 0.05 and |log2(foldchange)| > 1.

### 2.6. Gene Ontology (GO) and KEGG Annotation and Enrichment

The unigenes of *U. pumila* were mapped to *A. thaliana* gene IDs by sequence similarity searching against the genome of *A. thaliana* with an E-value cutoff of 1 × 10^−5^. The GO enrichment analysis for the differentially expressed genes (DEGs) in *U. pumila* samples were performed by the topGO package of R. The DEGs of *U. pumila* unigene IDs were transferred to the Arabidopsis TAIR locus IDs during the MapMan analysis. The software KOBAS were used to test the statistical enrichment of differential expression genes in KEGG pathways in *U. pumila* [44].

### 2.7. qRT-PCR Verification

The qRT-PCR was performed to verify the expression patterns revealed by the RNA-seq analysis. The purified RNA samples were treated with DNaseI and converted to cDNA using the PrimeScript RT Reagent Kit with gDNA Eraser (Takara, Dalian, China) according to the manufacturer’s procedures. A total of four up-regulated genes (Cluster_14363.17023, Cluster_14363.17552, Cluster_14363.18534, Cluster_14363.18706) and two down-regulated genes (Cluster_14363.19711, Cluster_14363.18394) were selected randomly for the qRT-PCR assay. An ortholog (Cluster-14363.8722) of the *A. thaliana* member of SAND family protein in *U. pumila* was used as a reference and to normalize the amount of template cDNA added in each reaction. Expression of five key genes of the “secondary walls synthesis” pathway and three genes of “phenylpropanoid and lignin biosynthesis” were tested in branch samples of UGu17 (Fast1, F1), UZuantian (Fast2, F2), U81-07 (Slow1, S1) and U82-39 (Slow2, S2) at spring (16 March 2020), summer (21 June 2020) and autumn (19 September 2020) by qRT-PCR analysis. Gene-specific qRT-PCR primers (21–24 bp) were designed using Premier 5.0 software (Table S4). qPCR was performed using SYBR Green qPCR Master Mix (DBI, Ludwigshafen, Germany) in an ABI7500 Real-Time PCR System (ABI, Waltham, MA, USA). Three replicates were performed, and the amplicons were used for melting curve analysis to evaluate the amplification specificity. Relative gene expression was quantified using the 2^−(ΔΔCt)^ method.

## 3. Results

### 3.1. Branches Phenotypic Traits

**Figure 1:**
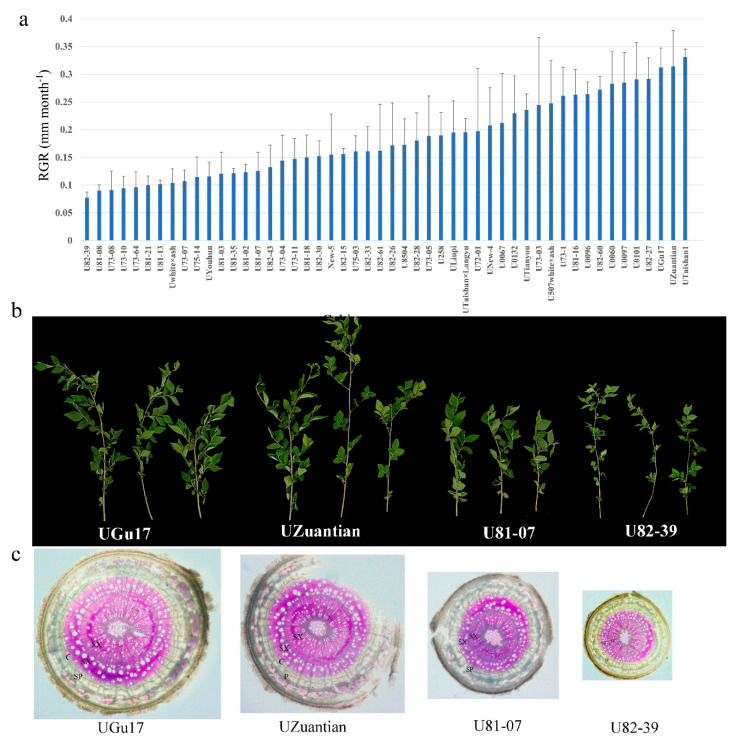
(**a**) The relative growth rates (RGR) of 50 elm cultivars from the Baiwa Forestry Centre. The variation range of RGR is 0.07–0.24 mm month^−1^, the average is 0.181 mm. The cultivar UTaishan1 has the highest BD (0.24) and the cultivar U82-39 has the highest BD (0.77). Error bars indicate SE. (**b**) The branch phenotypes of fast- (UGu17 and UZuantian) and slow-growing cultivars (U81-07 and U82-39). (**c**) Photomicrographs of branch cross-sections of fast- (UGu17 and UZuantian) and slow-growing cultivars (U81-07 and U82-39). C, cambium region; SP, secondary phloem; SX, secondary xylem.

The results of the conducted descriptive statistical analysis are shown for each studied cultivar (Figure S1a). The variation range of branch diameter is 0.65–2.36 mm, the average is 1.52 mm. The coefficient of variation (CV) value of branch diameter was 33.54%. The cultivar U73-03 has the highest BD (2.36 mm) and the cultivar U82-39 had the lowest BD (0.65 mm). Growth rates of cultivars were estimated by RGR. The variation range of RGR is 0.07–0.24 mm month-1, the average is 0.181 mm. The cultivar UTaishan1 had the highest BD (0.24) and the cultivar U82-39 had the highest BD (0.77) (Figure 1a). The phenotypic and comparative transcriptomic analyses were performed in *U. pumila* fast-growing cultivars UGu17 (2.13 mm) and UZuantian (2.21 mm) (Figure 1b,c) and slow-growing cultivars U81-07 (1.32 mm) and U82-39 (0.65 mm) (Figure 1b,c). Phenotypic observation showed that the thickness of secondary xylem of 2-year-old fast-growing branches (UGu17 and UZuantian) was significantly greater than that of slow-growing cultivars (U81-07 and U82-39) (Figure 1c).

### 3.2. Transcriptome Profiling of U. pumila

For branches, UGu17 and UZuantian were selected as representative cultivars of the fast-growing group with large BD values, U81-07 and U82-39 were used as representative cultivars of the slow-growing group with small BD values. After sequencing with the Illumina HiSeq X platform, a total of 20,176,918 to 24,632,251 pair-end reads were obtained from six samples with fast a branch growth rate and six samples with a slow branch growth rate (Table 1). De novo transcriptome assembly generated 60,784 unigenes, with an average length of 1204 nt and N50 of 1845. On average, 80.56% of the reads from twelve samples were mapped to the reference genome (Table 1).

### 3.3. Functional Annotations of Unigenes

**Figure 2:**
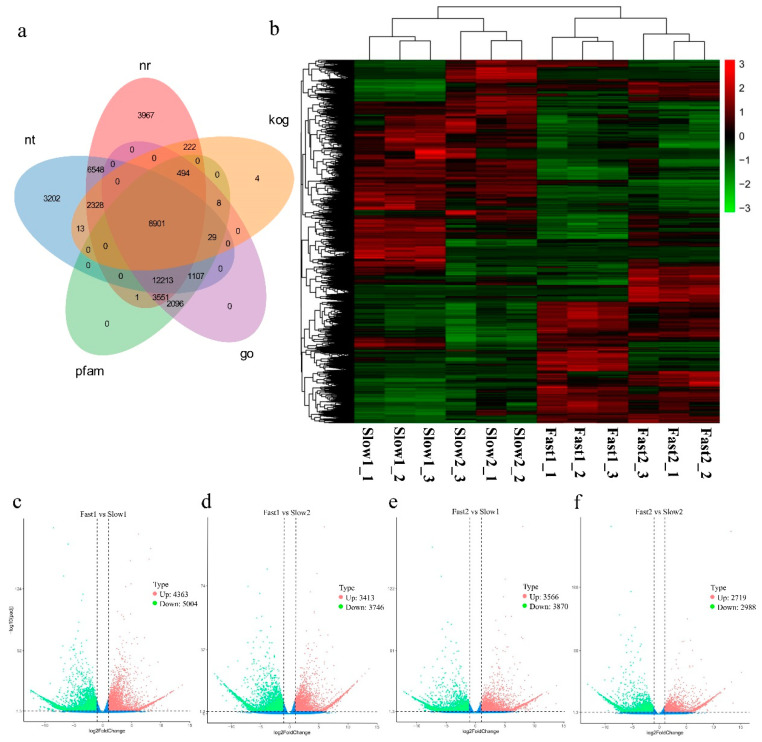
(**a**) Venn diagram of functional annotations of unigenes in nt (NCBI non-redundant protein sequences), nr (NCBI non-redundant protein sequences), kog (Clusters of Orthologous Groups of proteins), go (Gene Ontology) and pfam (Protein family) databases. (**b**) Heatmap of expression patterns in fast- (Fast1: UGu17 and Fast2: UZuantian) and slow-growing cultivars (Slow1: U81-07 and Slow2: U82-39) samples. (**c**–**f**) Expression patterns of differentially expressed genes (DEGs) identified between fast- and slow-growing cultivars. Red and green dots represent DEGs, blue dots indicate genes that were not differentially expressed. Totals of 9367 (Up = 4363, Down = 5004), 7159 (Up = 3413, Down = 3746), 7436 (Up = 3566, Down = 3870), 5707 (Up = 2719, Down = 2988) unigenes were filtered as dysregulated genes in comparisons between Fast1 vs. Slow1 (F1vS1), Fast1 vs. Slow2 (F1vS2), Fast2 vs. Slow1 (F2vS1), Fast2 vs. Slow2 (F2vS2) with the cutoff of padj < 0.05 and |log2(foldchange)| > 1.

Similarity searches by BLASTX were performed to annotate unigenes against different databases. All 60,784 (100%) unigenes were annotated in at least one database. A total of 38,225 (62.88%), 34,341 (56.49%) and 28,400 (46.72%) unigenes showed similarity to sequences in NR, NT and PFAM database with an E-value threshold of 1 × 10^−5^ (Figure 2a). A total of 28,399 (46.72%) unigenes were annotated in the GO database by Blast2GO v2.5 with an E-value cutoff of 1 × 10^−6^. A total of 30,916 unigenes of *U. pumila* were assigned to A. thaliana gene IDs for GO annotation mapping by BLASTX with an E-value cutoff of 1 × 10^−5^.

### 3.4. Differentially Expressed Genes (DEGs) Calculation

**Figure 3:**
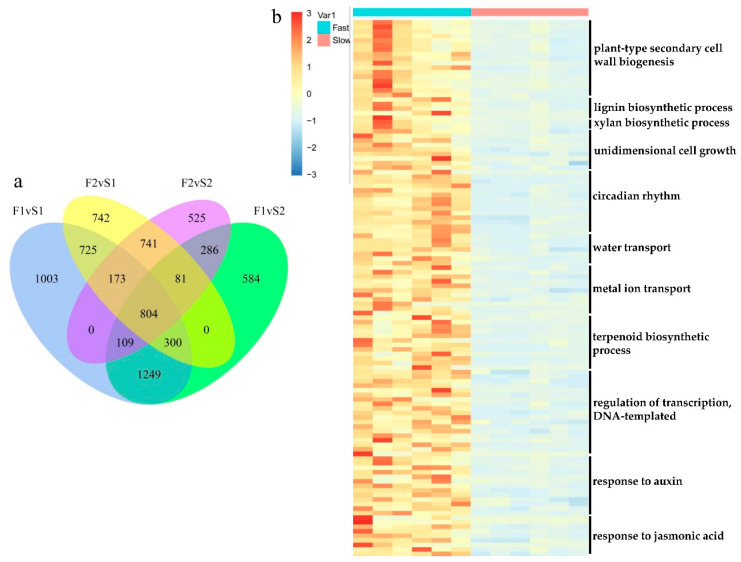
(**a**) The Venn analysis result of upregulated genes for fast-growing samples compared with slow-growing samples. There were 804 common up-regulated genes for fast samples compared with slow samples. Samples of Fast1 vs. Slow1 and Fast2 vs. Slow1 (F2vS1) had a relatively large number of characteristic up-regulated genes. (**b**) Expression patterns of 118 differentially expressed genes (DEGs) enriched in the key gene ontology (GO) biological processes (BPs).

The relative level of gene expression in *U. pumila* branches was evaluated by the FPKM values, which were calculated based on the uniquely mapped reads. Totals of 9367 (Up = 4363, Down = 5004), 7159 (Up = 3413, Down = 3746), 7436 (Up = 3566, Down = 3870), 5707 (Up = 2719, Down = 2988) unigenes were filtered as dysregulated genes in comparison of Fast1 vs. Slow1 (F1vS1), Fast1 vs. Slow2 (F1vS2), Fast2 vs. Slow1 (F2vS1), Fast2 vs. Slow2 (F2vS2) with the cutoff of padj < 0.05 and |log2(foldchange)| > 1 (Figure 2b–f, Table S1). Overlapping studies found that there were 804 common up-regulated genes for fast samples compared with slow samples, and the overlapping details were shown in Figure 3a. Samples of Fast1 vs. Slow1 and Fast2 vs. Slow1 (F2vS1) had a relatively large number of characteristics up-regulated genes.

### 3.5. Gene Ontology (GO) and KEGG Enrichment Result of DEGs

To uncover the potential pathways for branch growth in elm cultivars, the DEGs were characterized with GO databases. As a result, a total of 155, 150, 151 and 129 biological processes (BP) terms were enriched by the 4363, 3413, 3566 and 2719 up-regulated unigenes with a cutoff of *p*-value < 0.05 (Table S2). The comparison of four GO enrichment analyses indicated that “plant-type secondary cell wall biogenesis” (GO:0009834), “cell wall thickening” (GO:0052386), “flavonoid biosynthetic process” (GO:0009813) and “response to karrikin” (GO:0080167) were commonly enriched by genes up-regulated in fast samples. The heatmap of the expression pattern of the key genes in the representative over-expressed processes is shown in Figure 3b.

The KEGG pathways enriched by up-regulated unigenes were shown in Table S3 and top ten pathways are represented in Table 2. The KEGG pathway “Phenylpropanoid biosynthesis” (ko00940) was enriched by 60, 43, 46 and 25 up-regulated unigenes, “Flavonoid biosynthesis” (ko00941) was annotated by 11 to 25 over-expressed genes, and 11 to 18 up-regulated genes were annotated in the KEGG pathway “DNA replication” (ko03030) (Table 2 and Table S3). Besides, “Photosynthesis-antenna proteins” (ko00196) were enriched by genes commonly up-regulated in comparisons of F1vS1, F2vS1 and F2vS2 (Table 2 and Table S3).

### 3.6. Gene Expression Pattern and Functional Transition of Fast- and Slow-Growing Genotypes

**Figure 4:**
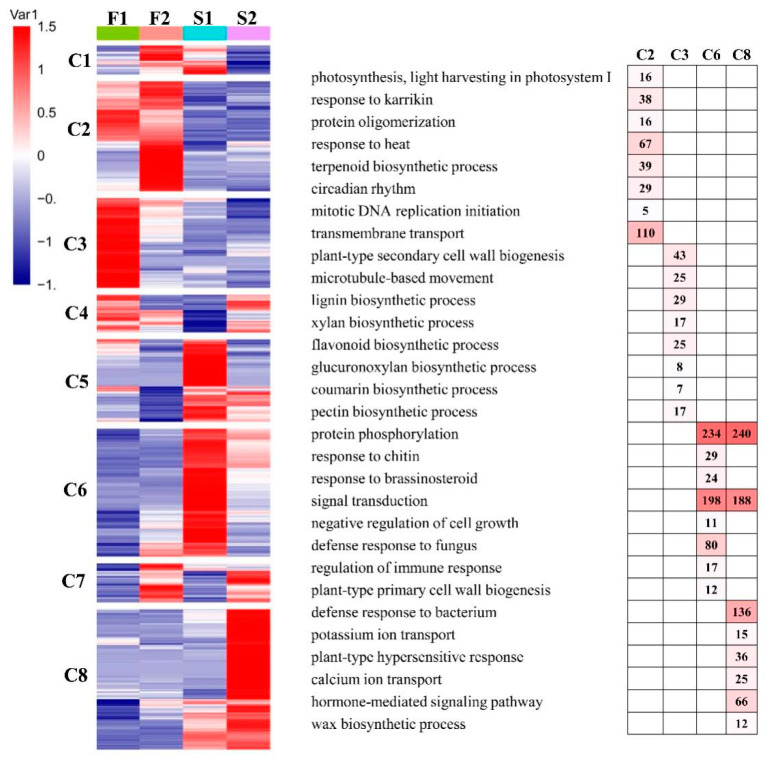
Gene expression pattern and functional transition of fast- and slow-growing genotypes. Expression patterns of 15,411 differentially expressed genes (DEGs) genes in eight DEG clusters. The number of genes in each cluster are shown on the right. Gene ontology (GO) enriched in eight DEGs clusters. Top eight significant categories (*p*-value < 0.05) are displayed.

To further provide insights into the functional transitions of fast- and slow-growing genotypes of elm, we clustered the union of DEGs (15,411 unigenes) into eight clusters using the euclidean distance clustering algorithm (Figure 4). The GO annotation was performed to assign genes to functional categories for each cluster (Figure 4). Genes belonging to cluster three (C3, 2110 genes) were mainly expressed in F1 (UGu17). This cluster contained a set of genes related to “plant-type secondary cell wall biogenesis”, “lignin biosynthetic process” and “lignin biosynthetic process”. Genes in cluster two (C2, 2583 genes) were synchronously upregulated in F2 (UZuantian). These genes function in “photosynthesis, light harvesting in photosystem I”, “protein oligomerization” and “terpenoid biosynthetic process”. Genes included in cluster six (C6, 3005 genes) were up-regulated in genotype S1 (U81-07) and participated in “protein phosphorylation”, “signal transduction”, and “defense response to fungus” (Figure 4). The genes in cluster eight (C8, 3291 genes) were highly expressed in S2 (U82-39) and represented by genes related to “signal transduction”, “defense response to bacterium”, and “hormone-mediated signaling pathway” (Figure 4).

### 3.7. Real-Time Quantitative PCR Validation

**Figure 5:**
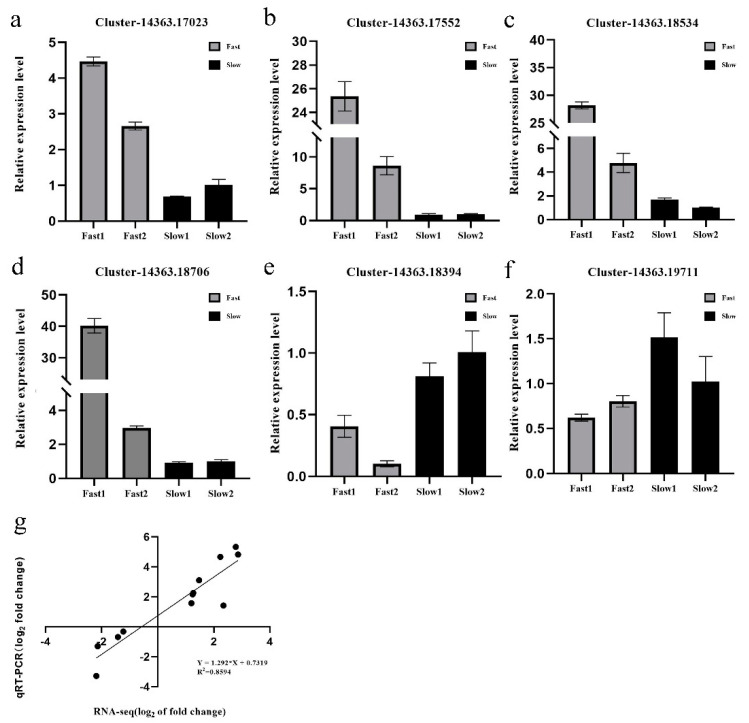
Validation of RNA-Seq data. Verification of the expression level of the selected eight DEGs from RNA−Seq data through RT-qPCR: (**a**) Cluster_14363.17023, (**b**) Cluster_14363.17552, (**c**) Cluster_14363.18534, (**d**) Cluster_14363.18706, (**e**) Cluster_14363.18394, (**f**) Cluster_14363.19711. Error bars indicate the standard error as mean + SD. The *x* axis represents the relative expression level, and the *y*-axis represents cultivars. (**g**) Correlation coefficient of gene expression between qRT−PCR analysis and RNA-Seq data.

To verify the RNA-Seq results, an alternative strategy was selected for the dysregulated unigenes. In total, four over- and two under-expressed unigenes were selected for validation by real-time quantitative PCR (qRT-PCR) using the same RNA samples that were used for RNA-Seq. Primers were designed to span exon-exon junctions (Table S4). In most cases, the gene expression trends were similar between these two methods, the correlation between the two sets of data was R^2^ = 0.859, the result was shown in Figure 5. For example, the homolog of the class I small heat-shock protein, Cluster-14363.17023, which was detected by RNA-Seq as up-regulated unigene in the fast-growing samples (Log2 fold change (L_2_fc) of F1vS1, F1vS2, F2vS1, F2vS2 = 2.069, 1.246, 3.170, 2.343), was also detected significantly over-expressed by the qRT-PCR method (Figure 5).

### 3.8. qRT-PCR Analysis for Secondary Walls and Lignin Biosynthesis-Associated Genes in Different Seasons

Information on the major gene expression variations that occur during branch growth has been predicted in elm fast- (UGu17 and UZuantian) and slow-growing cultivars (U81-07 and 11 U82-39) in the growth season; however, transcriptome analysis is limited to only summer. To provide more information about other seasons, the qRT-PCR analysis for key genes of the “secondary walls synthesis” pathway and three genes of “phenylpropanoid and lignin biosynthesis” were tested in branch samples of fast- and slow-growing cultivars in spring (16 March 2020), summer (21 June 2020) and autumn (19 September 2020). Most of the unigenes upregulated in the fast-growing cultivars screened by RNA_seq were discovered to be overexpressed in summer and other seasons by qRT-PCR (Figure S2a–i). However, the difference of gene expression in March or September was less than that in June. For example, fold change values of F1/S1 in VND1-Cluster-15866 were 2.27 (16 March 2020), 5.67 (21 June 2020) and 2.00 (19 September 2020). Besides, statistical analysis showed that the gene expression of samples in June was generally higher than that in spring and autumn.

## 4. Discussion

**Figure 6:**
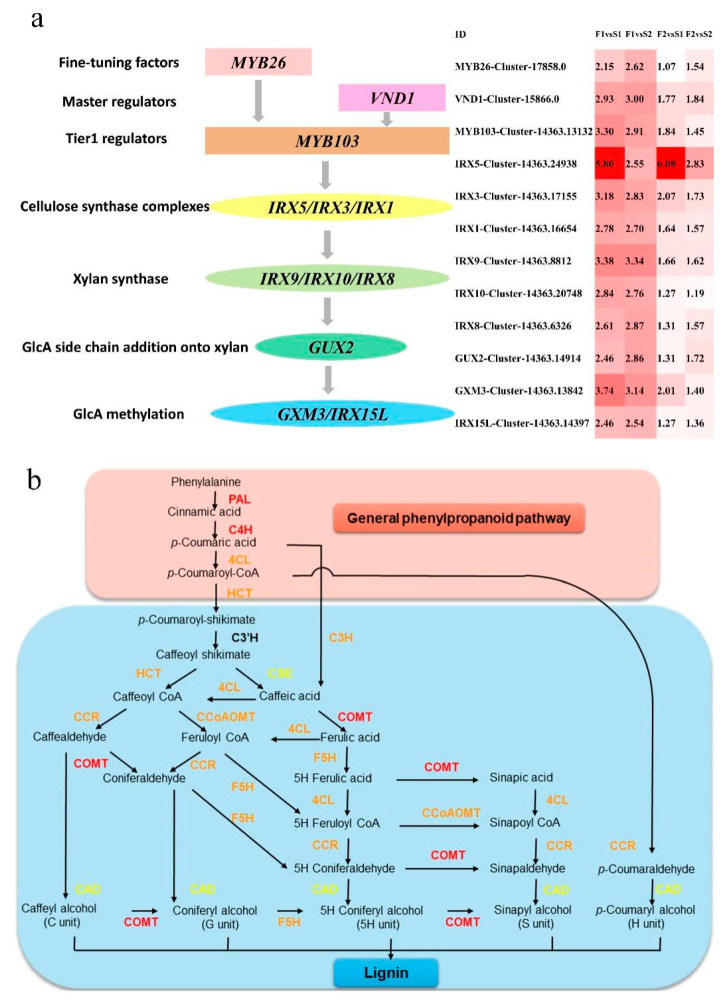
(**a**) Illustration of the predicted transcriptional regulating and secondary walls synthesis genes of branch growth in elm. The expression levels (Log2(FC)) of selected differentially expressed genes in fast- and slow-growing cultivars are shown on the right. A red color indicates that the gene is highly expressed in the branch samples. Log2(FC) means Log2() value of fold change for unigenes. (**b**) The biosynthesis pathway of the general phenylpropanoid and lignin. PAL, phenylalanine ammonia-lyase; TAL, tyrosine ammonia-lyase; C4H, cinnamate 4-hydroxylase; 4CL, 4-coumarate: CoA ligase; CCR, cinnamoyl-CoA reductase; HCT, hydroxycinnamoyl-CoA shikimate/Quinatehydroxycinnamoyltransferase; CCoAOMT, caffeoyl-CoA O-methyltransferase; F5H, ferulate 5-hydroxylase; CSE, caffeoyl shikimate esterase; COMT, caffeic acid O-methyltransferase; CAD, cinnamyl alcohol dehydrogenase. The color of the proteins indicates the expression of their coding genes in fast and slow growing branches: red indicates up-regulated in the four comparisons (F1vS1, F1vS2, F2vS1, F2vS2), orange indicates up-regulated in three comparisons, yellow indicates up-regulated in two comparisons.

The most important process in the growth of tree branches is the formation of vascular tissue. The procambium/cambium proliferation, vascular patterning, and xylem differentiation are regulated by plant hormones, transcriptional regulators, and peptide signaling, respectively. NAC domain TFs function as primary regulators in the biosynthesis of cellulose, hemicellulose, and lignin in xylary fibers [11,28,33,45]. The illustration of the predicted transcriptional regulating and secondary walls synthesis genes of branch growth in elm are shown in Figure 6a and Table 3. In Arabidopsis, the xylem vessel differentiation is positively regulated by *VND6* and *VND7* [46]. *VND1* to *VND5* function redundantly with *VND6* and *VND7* in vessel development [31]. In the present study, VND1 coding gene *VND1* (Cluster-15866.0, L2fc = 2.93, 3.00, 1.77, 1.84) was up-regulated in the fast-growing samples. A positive regulator MYB26 was found to be a primary regulator modulating the NAC domain [47]. The SCW deposition was enhanced when MYB26 was overexpressed. Furthermore, *MYB26* positively modulated the accumulation of SCWs. We found that the MYB26 homolog was over-expressed (Cluster-17858.0, L_2_fc = 2.15, 2.62, 1.07, 1.54) in the fast-growing elm branches. In the process of vascular development, xylem cells developed SCWs, which formed the biggest section of lignocellulosic biomass in plants. The over-expression of *VND1* and *MYB26* indicated that VND proteins regulated the biosynthesis of SCW in xylem vessels. It is one of the decisive factors for the growth of elm branches. A variety of other TFs serve as primary regulators downstream of the NAC and MYB domains [45,48]. The homolog of *MYB103* in elm was up-regulated in the fast-growing samples (Cluster-14363.13132, L_2_fc = 3.30, 2.91, 1.84, 1.45). This result indicated that cellulosic synthesis and enhanced SCW thickening in fibers were related to the thickening mechanism of elm branches.

The main components of SCWs include cellulose, xylan, glucomannan, and lignin. Cellulose synthase complexes synthesize cellulose in the plasma membrane [49,50]. In the present study, CESA homologs *IRX5* (Cluster-14363.24938, L2fc = 5.80, 2.55, 6.08, 2.83), *IRX3* (Cluster-14363.17155, L_2_fc = 3.18, 2.83, 2.07, 1.73), and *IRX1* (Cluster-14363.16654, L_2_fc = 2.78, 2.70, 1.64, 1.57) were over-expressed in fast-growing branches. As the second most abundant polysaccharide in SCWs of angiosperms, xylan plays a vital role in a structural network for secondary wall strength by forming twofold helical screw ribbons [7,51]. In elm, xylan synthase coding genes *IRX9* (Cluster-14363.8812, L_2_fc = 3.38, 3.34, 1.66, 1.62), *IRX10* (Cluster-14363.8812, L_2_fc = 5.80, 2.55, 6.08, 2.83), and *IRX8* (Cluster-14363.24938, L_2_fc = 5.80, 2.55, 6.08, 2.83) were up-regulated in the branches with coarser diameters. Besides, the over-expression of glucuronoxylan methyltransferases (GXMs) of DUF (domain of unknown function) 579 family *GXM3* (Cluster-14363.8812, L_2_fc = 3.38, 3.34, 1.66, 1.62) and *IRX15L* (Cluster-14363.8812, L_2_fc = 3.38, 3.34, 1.66, 1.62) indicated that GlcA methylation was enhanced in the fast-growing branches. These results suggested that cellulose amount, xylan synthesis, and SCW thickness were related to the thickening mechanism of elm.

As a significant metabolic process in plants, phenylpropanoid metabolism greatly contributes to plant development and plant-environment interplay [34]. The general phenylpropanoid pathway is composed of the first three steps of the phenylpropanoid pathway. The reactions in the general phenylpropanoid pathway are catalyzed by phenylalanine ammonia lyase (PAL), cinnamic acid 4-hydroxylase (C4H), and 4-coumarate-CoA ligase (4CL) [52]. In the present study, *PAL* (Cluster-14363.18746, L_2_fc = 3.04, 2.66, 1.86, 1.48), *4CL* (Cluster-14363.17413, L_2_fc = 2.94, 3.38, ns, 2.45) and *C4H* (Cluster-14363.19028, L_2_fc = 3.38, 3.03, 2.13, 1.79) coding genes were up-regulated in the fast-growing elm branch samples (Figure 6b, Table 3). These results indicated the general phenylpropanoid pathway was related to the thickening mechanism of elm branches. As one of the most important secondary metabolites, lignin is synthesized through the phenylalanine/tyrosine metabolic pathway in plant cells. In *Nicotiana tabacum*, hydroxycinnamoyl-CoA shikimate/quinate hydroxycinnamoyl transferase (HCT) is identified as the gateway enzyme of lignin biosynthesis [53]. We found that the homolog of *HCL* (Cluster-14363.5128, L_2_fc = 3.49,7.96, ns, 6.09) was over-expressed in the fast-growing elm branches. Besides, several vital enzymes, such as coumarate 3-hydroxylase C3H, caffeoyl shikimate esterase CSE, caffeate/5-hydroxyferulate 3-O-methyltransferase COMT, caffeoyl CoA 3-O-methyltransferase CCoAOMT, cinnamoyl-CoA reductase CCR, cinnamyl alcohol dehydrogenase CAD, fihydroflavonol 4-reductase DFR, and ferulate 5-hydroxylase F5H, in the lignin synthesis pathway were up-regulated in fast-growing branches of elm (Figure 6b, Table 3). These results indicated that lignin and its underlying pathway played a crucial role in the growth of elm branches. We speculated that lignin improved cell wall rigidity and hydrophobic properties and enhanced mineral delivery using the vascular bundles in fast-growing branches [17].

The transcriptional process and signaling pathways play a vital role in lignin synthesis and plant vascular tissue growth [33,45]. In the present study, many bZIP, C3H, Dof, and MYB TF family members were differentially expressed in the fast-growing branches. Moreover, five MYB TFs were over-expressed in the fast-growing elms. *MYB66* encoding a MyB-related protein-containing R2 and R3 repeats participated in root and hypocotyl epidermal cell fate determination in Arabidopsis [54]. The over-expression of its homolog (Cluster-14363.50, L_2_fc = 2.63, 2.07, 2.78, 2.22) in the fast-growing branches indicated its function in transcription during cell fate. *MYB105* functions in boundary specification in model plants [55]. The up-regulation of this gene (Cluster-16574.0, L_2_fc = 5.93, 3.14, 6.63, 3.85) in fast-growing elm branches indicated the role of organ boundary patterning and meristem initiation/maintenance in the development of elm branches. In Arabidopsis, *AtNAC2* is involved in lateral root development [56]. The expression pattern of this gene in the fast-growing branches indicated the function of this TF in ethylene and auxin signaling pathways during the thickening of elm.

The environmental factors affecting trees are climate, soils, topography, and biota. The soil microbial community is responsible for most nutrient transformations in soil, regenerating minerals that limit tree growth [57,58,59]. In this study, immune responding genes were differentially expressed in the slow-growing genotypes, as “defense response to fungus” enriched by 80 over-expressed unigenes in S1 (U81-07) and “defense response to bacterium” included 136 up-regulated unigenes in S2 (U82-39). However, the unigenes of two fast-growing genotypes did not enrich in the process of soil microorganisms’ response. The above results indicated that the microorganisms in the source environment of slow growing strains may be different from that of Baiwa Forest farm. Therefore, there is a response to environmental microorganisms in the process of tree growth and development, resulting in slow growth.

Although we identified the molecular underpinning of biologically active substances in growing elm branches through sequencing and bioinformatics methods, we did not test any of the above-mentioned genes, which remained a limitation of our current investigation. Collectively, it is necessary to carry out functional analyses to further identify the functions of phytonutrient-associated genes and pathways in elm thickening.

## 5. Conclusions

In the present study, phenotypic and comparative transcriptomic analyses were performed in Elm fast- (UGu17 and UZuantian) and slow-growing cultivars (U81-07 and U82-39). Phenotypic observations showed that the thickness of secondary xylem of 2-year-old fast-growing branches was greater compared with slow-growing cultivars. Comparative transcriptome analysis predicted that many pathways were involved in vascular development and transcriptional regulation in elm, such as “plant-type secondary cell wall bio-genesis”, “cell wall thickening”, and “phenylpropanoid biosynthesis”. NAC domain transcriptional factors and their master regulators, cellulose synthase catalytic subunits, xylan synthesis, and secondary wall thickness were supposed to function in the thickening mechanism of elm branches. Our results indicated that the general phenylpropanoid pathway and lignin metabolism had vital functions in the growth of elm branches.

## Figures and Tables

**Table 1:** Summary of the mapping of transcriptome reads to the reference sequence.

Sample Name	Total Reads	Total Mapped	Mapping Ratio
Fast1_1	49,014,302	39,324,430	80.23%
Fast1_2	41,483,608	33,367,760	80.44%
Fast1_3	41,955,680	33,679,770	80.27%
Fast2_1	41,945,930	33,784,144	80.54%
Fast2_2	40,140,204	32,087,136	79.94%
Fast2_3	42,014,884	33,631,348	80.05%
Slow1_1	44,732,848	35,697,784	79.80%
Slow1_2	42,697,528	34,428,142	80.63%
Slow1_3	40,034,844	32,328,242	80.75%
Slow2_1	42,918,790	34,843,800	81.19%
Slow2_2	41,464,616	33,715,910	81.31%
Slow2_3	41,702,398	34,017,546	81.57%

**Table 2:** The top KEGG pathways enriched by upregulated genes.

KEGG Pathway	F1VS1	F1VS2	F2VS1	F2VS2
Phenylpropanoid biosynthesis	1.27 × 10^−11^	9.28 × 10^−9^	6.83 × 10^−9^	7.54 × 10^−4^
Flavonoid biosynthesis	6.02 × 10^−10^	3.52 × 10^−11^	2.25 × 10^−3^	1.73 × 10^−4^
Stilbenoid, diarylheptanoid and gingerol biosynthesis	2.83 × 10^−6^	6.41 × 10^−6^	2.46 × 10^−2^	-
Photosynthesis-antenna proteins	4.91 × 10^−4^	-	4.42 × 10^−6^	2.01 × 10^−4^
DNA replication	6.62 × 10^−4^	5.46 × 10^−5^	1.16 × 10^−3^	3.95 × 10^−3^
Starch and sucrose metabolism	2.47 × 10^−3^	-	4.54 × 10^−3^	
Diterpenoid biosynthesis	2.89 × 10^−3^	2.62 × 10^−2^	1.85 × 10^−4^	1.10 × 10^−2^
Linoleic acid metabolism	3.87 × 10^−3^	1.97 × 10^−3^	3.21 × 10^−2^	2.82 × 10^−2^
Plant hormone signal transduction	4.89 × 10^−3^		3.52 × 10^−5^	-
Phenylalanine metabolism	4.90 × 10^−3^	7.28 × 10^−5^	3.42 × 10^−3^	-

**Table 3:** The fold change ratio of genes in several key vascular development and transcriptional regulation pathways.

Process	Speculative Function	Unigene ID	F1vS1	F1vS2	F2vS1	F2vS2
plant-type secondary cell wall biogenesis	cellulose synthase	Cluster-14363.16654	2.781	2.704	1.643	1.570
		Cluster-14363.17155	3.181	2.834	2.070	1.726
		Cluster-14363.24938	5.799	2.547	6.081	2.832
	Exostosin	Cluster-14363.20748	2.841	2.757	1.268	1.185
	fasciclin-like arabinogalactan protein	Cluster-14363.18562	2.603	3.123	1.765	2.289
		Cluster-14363.14849	2.962	3.642	1.753	2.433
		Cluster-14363.25117	2.656	2.678	1.524	1.547
		Cluster-14363.6739	4.230	4.432	2.344	2.552
	glucuronoxylan 4-O-methyltransferase	Cluster-14363.13842	3.744	3.140	2.008	1.404
	glucuronyltransferase	Cluster-14363.14914	2.455	2.865	1.309	1.720
	glycosyl transferase	Cluster-14363.8812	3.382	3.340	1.658	1.617
	laccase	Cluster-14363.9138	3.477	3.514	1.736	1.774
	MYB transcription factor	Cluster-14363.13132	3.301	2.909	1.843	1.452
		Cluster-17858.0	2.152	2.620	1.069	1.538
	NAC transcription factor	Cluster-15866.0	2.925	2.997	1.766	1.845
	phytochelatin synthetase	Cluster-14363.17226	3.718	3.903	3.050	3.245
	xylan synthesis	Cluster-14363.14397	2.455	2.537	1.274	1.359
lignin biosynthetic process	chitinase	Cluster-14363.19471	3.163	2.963	1.626	1.427
	interfascicular fibers synthesis	Cluster-14363.13437	2.402	3.274	2.872	3.745
	laccase	Cluster-14363.7596	4.162	3.920	2.297	2.055
	O-methyltransferase	Cluster-14363.16741	3.258	2.758	2.537	2.039
xylan biosynthetic process	galacturonosyltransferase	Cluster-14363.6326	2.609	2.866	1.306	1.566
	synthesis and deposition of secondary wall cellulose	Cluster-14363.15500	3.517	3.539	2.184	2.206
unidimensional cell growth	actin monomer-binding protein	Cluster-14363.20463	1.884	2.078	1.727	1.920
	Alpha-Expansin	Cluster-14363.17330	4.839	2.400	4.704	2.264
	cell differentiation	Cluster-14363.13311	2.983	2.661	1.934	1.618
	expansin A6	Cluster-14363.28593	1.493	1.715	1.113	1.336
	Immunoglobulin E-set	Cluster-14363.18373	4.244	2.924	2.466	1.144
	key turgor pressure regulator in plant cells	Cluster-14363.35635	1.745	1.599	1.174	1.027
	plasma-membrane associated cation-binding protein	Cluster-14363.13070	2.431	2.956	1.436	1.961
water transport	ABC transporter	Cluster-14363.32034	1.416	2.427	1.937	2.949
	plasma membrane intrinsic protein	Cluster-14363.15480	1.787	1.351	1.987	1.553
		Cluster-14363.16016	2.476	2.960	2.677	3.161
		Cluster-14363.19969	2.378	2.143	2.477	2.242
		Cluster-14363.20258	1.136	1.286	1.258	1.410
	water and urea channels	Cluster-14363.8769	4.830	3.982	4.473	3.620
regulation of transcription, DNA-templated	Auxin inducible protein	Cluster-13847.0	1.559	1.218	1.797	1.455
	Auxin-responsive gene	Cluster-14363.35167	1.811	1.168	2.146	1.509
	B-box type zinc finger protein	Cluster-14363.31847	3.759	2.030	3.805	2.075
	bHLH DNA-binding protein	Cluster-14363.10371	3.195	2.068	3.374	2.250
		Cluster-14363.388	1.248	2.707	1.614	3.076
	C2H2 transcription factor	Cluster-14363.17522	2.245	1.967	1.666	1.391
		Cluster-14363.19420	2.797	3.476	2.111	2.800
		Cluster-14363.25933	1.883	1.732	1.247	1.098
	Dof transcription factor	Cluster-14363.5357	1.185	1.278	1.471	1.570
	multiprotein bridging factor	Cluster-14363.21135	1.831	2.109	2.962	3.240
	MYB transcription factor	Cluster-14363.31703	5.646	3.215	5.811	3.389
		Cluster-14363.50	2.631	2.067	2.778	2.216
		Cluster-16574.0	5.926	3.140	6.629	3.849
		Cluster-17031.0	1.934	1.959	1.801	1.827
		Cluster-17693.0	5.932	5.833	5.440	5.353
	zinc knuckle protein	Cluster-14363.23170	1.345	1.180	1.644	1.483
response to auxin	Auxin efflux carrier protein	Cluster-14363.23375	2.303	1.763	2.336	1.793
		Cluster-14363.23376	1.570	1.270	1.796	1.501
	modulator of auxin signaling	Cluster-14363.24574	1.570	2.781	1.064	2.279
	regulates vesicle trafficking	Cluster-14363.13767	1.350	1.129	2.224	2.006
	regulator of cellular auxin efflux	Cluster-14363.4717	2.327	1.505	2.275	1.457
response to jasmonic acid	controls the balance between salicylic acid and jasmonic acid signaling	Cluster-14363.16305	2.509	2.540	1.252	1.294
	NAC transcription factor	Cluster-18724.0	2.013	1.488	2.351	1.834
	required for wound-induced jasmonic acid accumulation	Cluster-14363.17181	2.753	2.264	2.969	2.478
	response to abscisisc acid and jasmonic acid	Cluster-16707.0	7.905	7.813	4.921	4.835
	vacuole formation	Cluster-14363.19730	3.595	3.708	2.602	2.710
	wound- and methyl jasmonate-induced secondary metabolism	Cluster-14363.28007	2.998	1.359	2.978	1.338

## Data Availability

All genetic data have been submitted to the NCBI Sequence Read Archive (SRA) database (https://submit.ncbi.nlm.nih.gov/subs/sra, accessed on 1 May 2022), PRJNA785411 for *U. pumila*.

## Supplementary Materials

The following are available online at https://www.mdpi.com/article/10.3390/biology11050711/s1, Table S1: The dysregulated gene list in comparisons of Fast1 vs. Slow1 (F1vS1), Fast1 vs. Slow2 (F1vS2), Fast2 vs. Slow1 (F2vS1), Fast2 vs. Slow2 (F2vS2), Table S2: The biological processes (BP) terms enriched by the up-regulated unigenes, Table S3: The KEGG pathways enriched by the up-regulated unigenes, Table S4: The real-time quantitative PCR (qRT-PCR) primer sequences. Figure S1: (a) The branch diameter of 50 elm cultivars from the Baiwa Forestry centre. The variation 191 range of branch diameter is 0.65–2.36 mm, the average is 1.52 mm. The coefficient of variation (CV) 192 value of branch diameter was 33.54%. The cultivar U73-03 has the highest BD (2.36 mm) and the 193 cultivar U82-39 has the highest BD (0.65 mm). (b) For each cultivar, three trees in good condition were used for the measurement. Three branches were measured randomly from the selected tree. Basal diameter (BD) of the internode was determined as the geometric mean of two measurements taken in perpendicular directions to 0.1 mm with digital calipers at the midpoint of the internode. Figure S2: Expression of five key genes of “secondary walls synthesis” pathway and three genes of “phenylpropanoid and lignin biosynthesis” were tested in branch samples of UGu17 (Fast1, F1), UZuantian (Fast2, F2), U81-07 (Slow1, S1) and U82-39 (Slow2, S2) at spring (March 16, 2020), summer (June 21, 2020) and autumn (September 19, 2020) by qRT-PCR analysis: (a) VND1-Cluster-15866.0, (b) MYB103-Cluster-14363.13132, (c) IRX3-Cluster-14363.17155, (d) IRX9-Cluster-14363.8812, (e) GXM3-Cluster-14363.138424, (f) IRX15L-Cluster-14363.14397, (g) PAL-Cluster-14363.26992, (h) 4CL-Cluster-14363.17413, (i) COMT-Cluster-14363.16741. Error bars indicate the std error. The *x* axis represents the relative expression level, and the *y*-axis represents three sampling seasons. The statistical differences between gene expression in Jun and Mar/Sep samples were analyzed by one-way anova. ***/### indicates *p*-value < 0.001, **/## indicates *p*-value < 0.01, */# indicates *p*-value < 0.05, ns means not significant.
