# Supplementary material for: Comparative Transcriptome Analysis Reveals Candidate Genes and Pathways for Potential Branch Growth in Elm (Ulmus pumila) Cultivars
Source: Biology (Basel). 2022 May 6;11(5):711. doi: 10.3390/biology11050711 (PMC9139171; doi:10.3390/biology11050711)
Supplement: Supplementary file 1 [file biology-11-00711-s001.zip › biology-1691440-supplementary/Supplementary Files/biology-1691440-Supplementary.pdf]

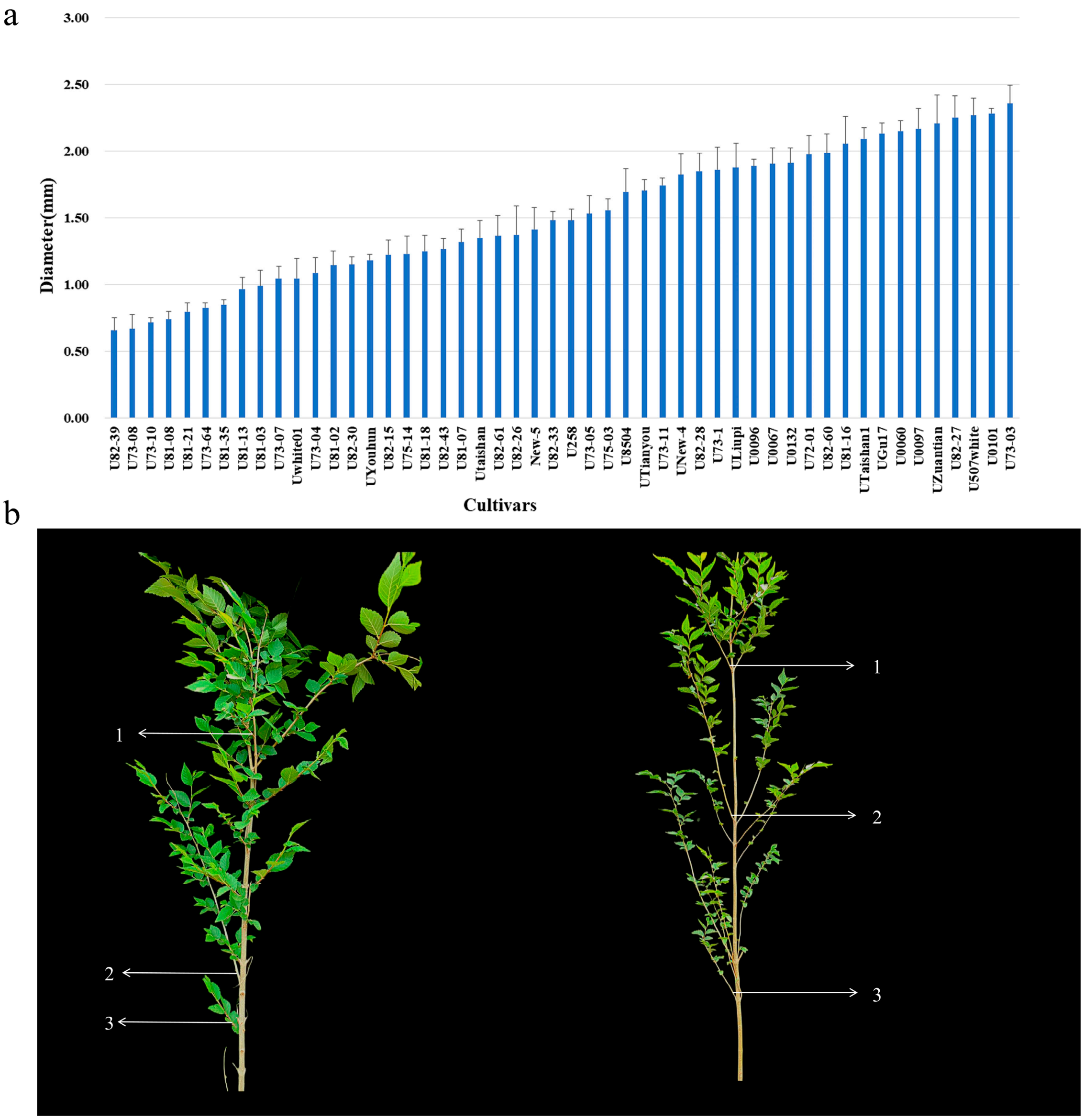

Figure S1. (a) The branch diameter of 50 elm cultivars from the Baiwa Forestry centre. The varia-tion 191 range of branch diameter is 0.65–2.36 mm, the average is 1.52 mm. The coefficient of variation (CV) 192 value of branch diameter was 33.54%. The cultivar U73-03 has the highest BD (2.36 mm) and the 193 cultivar U82-39 has the highest BD (0.65 mm). (b) For each cultivar, three trees in good condition were used for the measurement. Three branches were measured ran-domly from the selected tree. Basal diameter (BD) of the internode was determined as the geo-metric mean of two measurements taken in perpendicular directions to 0.1 mm with digital cal-ipers at the midpoint of the internode. .

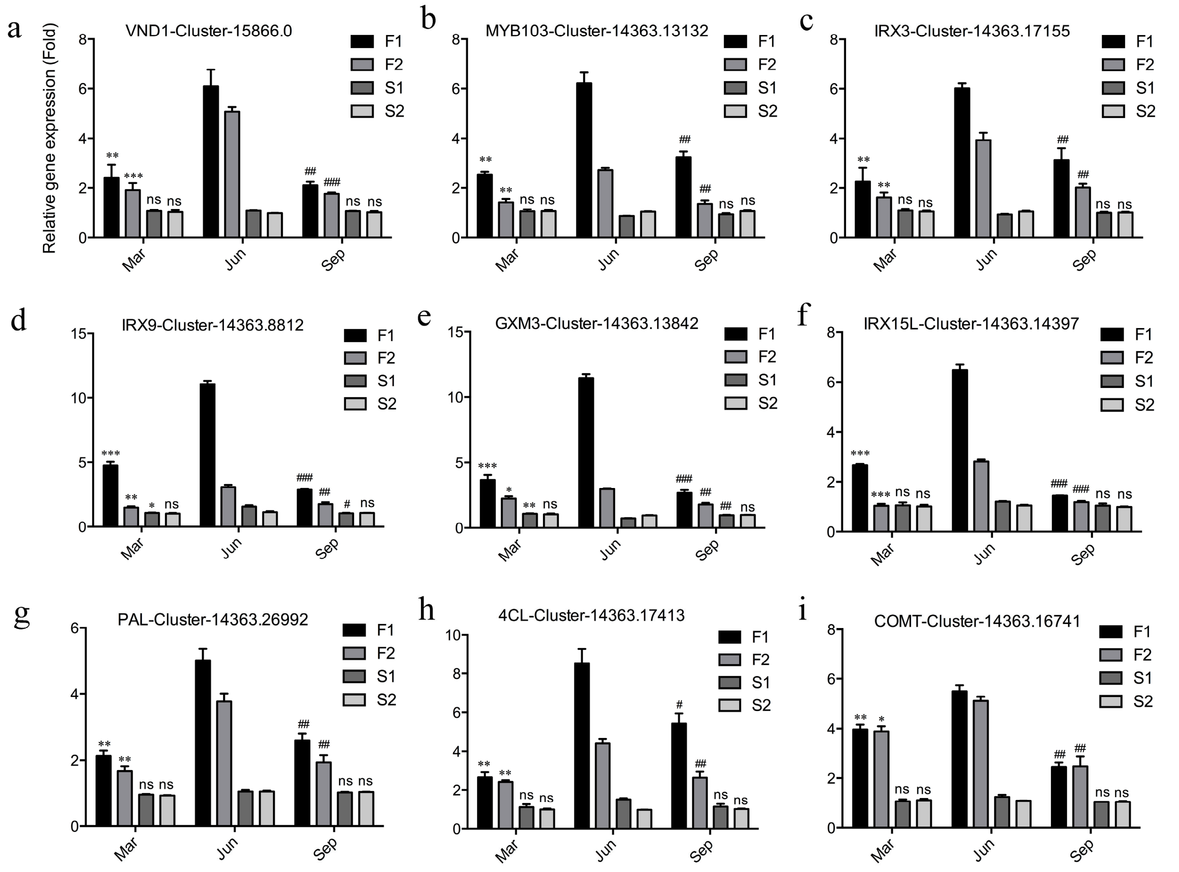

Figure S2. Expression of five key genes of "secondary walls synthesis" pathway and three genes of "phenylpropanoid and lignin biosynthesis" were tested in branch samples of UGu17 (Fast1, F1), UZuantian (Fast2, F2), U81-07 (Slow1, S1) and U82-39 (Slow2, S2) at spring (March 16, 2020), summer (June 21, 2020) and autumn (September 19, 2020) by qRT-PCR analysis: (a) VND1-Cluster-15866.0, (b) MYB103-Cluster-14363.13132, (c) IRX3-Cluster-14363.17155, (d) IRX9-Cluster-14363.8812, (e) GXM3-Cluster-14363.138424, (f) IRX15L-Cluster-14363.14397, (g) PAL-Cluster-14363.26992, (h) 4CL-Cluster-14363.17413, (i) COMT-Cluster-14363.16741. Error bars indicate the std error. The x axis represents the relative expression level, and the y-axis repre-sents three sampling seasons. The statistical differences between gene expression in Jun and Mar/Sep samples were analyzed by one-way anova. ***/### indicates p_value < 0.001, **/## indi-cates p_value < 0.01, */# indicates p_value < 0.05, ns means not significant.
